# Some Modern Aspects of Preventive Medicine

**Published:** 1900-12

**Authors:** David Samuel Davies

**Affiliations:** President


The Bristol

Medico-Chirurgical Journal.

DECEMBER, 1900.

### SOME MODERN ASPECTS OF PREVENTIVE MEDICINE.

The Presidential Address, delivered on October 10th, 1900, at the Opening of the Twenty-seventh Session of the Bristol Medico-Chirurgical Society.

BY

David Samuel Davies, M.D. Lond., D.P.H., President.

At a first glance there would seem to be a wide gulf separating the practice of Public Health from that of the twin crafts of Medicine and Surgery. And yet, on closer examination, we find the distinction in many points more imaginary than real; for, though the absolute practice of Public Health excludes participation in active medical or surgical work, those more recent advances in pathological mycology, which are indissolubly associated with the progress of the scientific attitude toward the control of disease, have at the same time not only helped to perfect the medical recognition and treatment of diseased conditions, but have actually rendered possible the evolution of modern surgical methods. A yet closer tie has been established by the direct help which laboratory methods have of recent years afforded towards the discrimination of diseases, a help which further progress should render still more definite and valuable, and one which has been generously and ungrudgingly recognised by the profession at large.

To such generous recognition of as yet small services I ascribe the graceful compliment which places me in your presidential chair, a position which I feel I hold in trust on behalf of the Public Health service, with a full and sincere appreciation of its responsibility and its dignity.

The *problem of causation* in regard to communicable diseases has in every age been of profound interest; but in no branch of medicine is to be found a more startling contrast than that which exists between the ancient and the modern attitude towards contagion. Amongst the ancients there is little doubt that plagues and pestilences were all classed together as of divine origin. Forms and ceremonies were used to avert disaster, temples and statues were erected to offended deities, the diseases themselves were deified; in later ages festivals were held, days of mourning decreed, and churches founded to appease divine wrath. In the absence of accurate knowledge of the pathology of contagion, this attitude is perfectly intelligible. Darwin points out that there is no exception to the rule that every organic being naturally increases at so high a rate that, if not checked, the earth would soon be covered by the progeny of a single pair. The epidemic spread of communicable disease dependent on parasitic organisms tends, in spite of the restrictive influences of environment, individual insusceptibility, or other opposing causes, to assume, and if not purposely checked ordinarily does assume, a geometrical rate of increase. If the ratio of increase is as little as twofold, the eighth term of such a series commencing with one case would be 128; if the ratio is threefold, the eighth term would be 2,187; whereas if the ratio is sixfold (a ratio I have observed in a commencing small-pox outbreak checked at this exact point), the eighth term of the series of an absolutely unfettered outbreak would have reached the large total of 279,936 cases. It is the old story of the nails in the horse-shoes.

The earlier terms of a geometrical progression are comparatively small; thus, considering the fourfold ratio of increase, the first eight consecutive terms are—

1, 4, 16, 64, 256, 1,024, 4,096, 16,384.

A disease introduced amongst a susceptible population, and spreading at this rate, might at first attract little notice, until the deaths began to be so numerous as to cause alarm. By this time the accumulation of cases would be so great as to obscure the rise and progress of the epidemic by natural means. "The threads of connection of case with case are inextricably entangled, and practically lost. There are hundreds or thousands of sick people, and the effect is so universal that the public lose their grasp of and confidence in the sufficiency of the cause to which science or intelligent knowledge firmly adheres. It is then we hear of epidemic constitutions, pandemic waves, and all those mysterious and unprofitable conceptions which are begotten of ignorance and terror in the human mind."[1] The correct view, even though the threads are lost, is to look upon every epidemic as invariably having begun with one case. Each case in the series possesses similar potentialities of increase, and all preventive measures must rightly be directed against the individual cases, the units of the disease.

The establishment of the causal connection between certain organisms and special diseases, in accordance with Koch's postulates, has been necessary to place the modern view of the communicable diseases upon a solid foundation; but it is interesting to note how shrewdly it had been guessed from simple observation and argument from analogy.

Up to the time of the devastating epidemics of the 14th century[2] no advance was made, and pestilence was still ascribed to supernatural agencies; but the experiences of this century seem to have stimulated thought, and to have started the modern idea of contagion, indicated by the establishment of lazarettos and the institution of quarantine. In[3] 1546 Girolamo Fracastoro, the Italian physician, first enunciated this doctrine

[1] Russell, *Lectures on the Theory and General Prevention and Control of Infectious Diseases*, 1879, p. 71.

[2] The immense effect these epidemics had upon the relationship between landlord and tenant, resulting almost in "the emancipation of the surviving serfs," and other economic effects, are dealt with in Prof. Thorold Rogers's *Six Centuries of Work and Wages*, 1884; *The Economic Interpretation of History*, 1888.

[3] Sykes, *Public Health Problems*, 1892.

as to plague, asserting that a specific contagion was exhaled by the bodies of infected persons, spreading only short distances by the air, but clinging so tenaciously to various objects as to be carried to unlimited distances and to be capable of infecting whole cities. Accurate knowledge was of course impossible until the successive impulses afforded by microscopic advance, by differentiation of the types of fever, by establishment of the cellular theory of plants and animals, and by the study of inflammatory processes, conjoined with much careful observation of diseased conditions and of the influences affecting them, and with more intelligent study of immediate transmission of disease from person to person, and of mediate transmission through water, air, or soil, paved the way for the discovery of actual causes, and resolved the study of communicable disease into a study of the life-history of parasitic organisms. Between the animal parasites of man and the micro-organismal parasites associated with his specific fevers, there are so many points of analogy that the consideration of the life history of the former may well help to elucidate the less obvious phenomena presented by the latter. Leuckart's views as to the evolution of parasitic species have been ably dealt with by Payne, and are of considerable interest in relation to the specific diseases. The argument runs thus[1]: —

In considering the question of parasitism, we must allow that any parasite must have been evolved from some free living form. In many parasites no relationship with known free living forms can be traced, survival of the parasitic form being due to the greater advantages of its habits. The Nematoid intestinal worms, however, possess near allies in the free water-living Nematoids, feeding on decomposing organic matter. Their food supply is precarious, and drying of the stagnant pools they inhabit threatens them with extinction. Now such worms must often be carried into the intestines of larger animals drinking the water, and if able to accommodate themselves to the conditions of existence there, they gain great

[1] Condensed from Dr. J. F. Payne's "Specific Diseases considered with Reference to the Laws of Parasitism," *St. Thomas's Hosp. Rep.*, 1892, xx. 59; *Manual of General Pathology*, 1888.

economical advantages, including large supplies of food and an equable temperature, both conducive to the heightened fertility characteristic of parasitic forms. If the pools dry up, the parasite accompanies the animal to fresh drinking places, and secures fresh facilities for development, when the cycle may be repeated. By natural selection the individuals suited to an intestinal life would remain, and the unfit be eliminated—thus a species would result, spending its adult life as an intestinal parasite, and its embryonic life only in the water. Similarly it may be assumed that the micro-organismal parasites, each after its kind, are derived from free living forms, the line of descent to be traced through those forms inhabiting the medium from which the animal body derives each class of parasite—from water, from air, or from soil. The parasitic habit having been established, the conditions favouring the continuance of the species are regulated by the laws of parasitism, of *entrance*, *migration*, and *exit*, each of these being such as to secure the successive passage of the parasite into the next proper medium, or the next necessary host.

The law of *entrance* is, in the case of animal parasites generally simple, requiring only the habit of aquatic life, so that they may be ingested with food or water. The purely intestinal parasite remains in the intestine, but certain species migrate to other organs more suitably situated for the continuance of the species.

The bacillus of enteric fever and the spirillum of cholera are both adapted for aquatic life, actively mobile by flagella, nearly allied to species common in foul liquids, marshes, or stagnant pools, and, so far as we know, exclusively human in their parasitic life.

It requires, therefore, says Payne, no great stretch of the imagination to suppose that such diseases were originally developed from an aquatic free species living in foul liquids, and that selected individuals became adapted to living in the intestines, where their multiplication would be favoured by high temperature, and by this adaptation they would be carried from one place to another, and thus when discharged from the bowels would be able to colonise fresh localities. The fact that they are exclusively human suggests, too, that their evolution has been connected with human systems of excrement disposal, and has become indissolubly associated with these arrangements. As to one of these diseases—enteric fever—Sir William MacGregor[1] remarks on its absence in British New Guinea, in West Africa, and till recently in Fiji, and suggests that it is a by-product of civilisation. The mode of entry of cholera and enteric fever germs is by water, to which they may return, and in which, or in moist soil organically polluted, they appear to live their part term of life as saprophytes.

In obedience to the second and third laws, those of *migration* and *exit*, embryos find their way to some part of the body offering the best opportunities for the continuance of their species. For example, the tapeworm form of the human tænia echinococcus, which is peculiar to the dog and the wolf, discharges its final mature proglottis, which may contain as many as 5000 ova, on to the surface of the ground, whence it is taken up into its next host, normally some herbivorous animal, the echinococcus being apparently accidental in man. Next occurs the curious migration of the six-hooked embryos set free, usually to the liver, which is probably the first point of attack when the herbivorous host is eaten by dog or wolf, in which the tapeworm form again develops. Similarly, human tapeworms pass their embryonic stage in oxen or pigs, in which they find their way to the muscular tissues, to be devoured in turn by carnivora or by man, in whom they re-assume the tapeworm stage.

A further example illustrating the adoption of habits well fitted to ensure the continuance of the species is found in the life history of the Pentastoma tænioides, which in its mature form hangs in the frontal sinuses and nostrils of dogs and wolves; passing thence with the nasal mucus on to plants, it is eaten by herbivorous animals, usually rabbits, in which it passes directly to the liver. The dog or wolf attacks first the liver, plunging his nose into it, the embryo becomes attached to the nostrils, and so the cycle is continued.

In the case of vegetable parasites, there are many analogies

[1] *Brit. M. J.*, 1900, ii. 979.

with the phenomena of animal parasitism. Some micro-organisms produce only local trouble, but others at once migrate to situations favourable for their continuance, the migration being accompanied by enormous increase in numbers. The acute exanthemata furnish a notable example; the parasitic microbes received by inhalation migrate so as to reach the surface of the skin, where they set up inflammatory processes of a kind to secure their being thrown off into the air, and so to favour the continuance of the species; and this cutaneous excretion of infection takes place by whatever channel the virus is received into the body, whether the small-pox is inoculated or the scarlet fever received with milk.

A very important factor in connection with the continuance of the parasitic micro-organismal diseases is the degree of infectivity in relation to the duration of the disease: the acute specific fevers are in general short-lived, and to secure continuance must present an abnormally high potentiality of infection; the diseases of slight infectivity, such as tubercle and leprosy, are characterised by long duration, which, together with a high rate of multiplication, tends to the preservation of the species.

It is an obvious advantage to a parasitic species to have the power of continuing its existence in more than one kind of animal. In this respect cholera and enteric fever are at a disadvantage, as they are distinctively human, and if the immediate or mediate transmission from man to man be inhibited, they must rely only on their powers of saprophytic existence. Small-pox has also only limited powers of spread amongst animals other than man, and these are of such kind that they have been turned to man's advantage. Scarlet fever, tubercle and diphtheria, however, introduce us to a class of disease whose prevalence amongst the lower animals has distinctively led to some distribution of the human disease; and plague in its turn presents the most notable instance of an ability to exist in epidemic form simultaneously in man and a wide group of animals often associated with him. The rat, mouse, monkey, bandicoot, squirrel, marmot, guinea-pig, and rabbit are all liable to contract plague under natural conditions, and in such fashion, especially in regard to the rat, as to materially assist or precipitate the spread of an epidemic amongst men. Koch, indeed, appears to consider that plague is primarily a rat disease, its occurrence in the human subject being secondary or accidental. This introduces a difficulty not met with in our other epidemic diseases, and demands more than the fitful interest hitherto accorded to the subject.

The chances of survival in any parasite are generally very slight, and, were not the rate of production enormous and the evolved habits favourable, the species would tend to die out. Cholera in unfavourable climates, where no fresh supplies of spirilla able to take on the intestinal habit are available, and small-pox under the double disadvantages of vaccination and isolation, do become locally extinct. Tubercle is an example of an organism which maintains its existence by extreme multiplication, long continuance, and selection of special sites for its elimination. By whatever means tubercle is introduced, it ultimately, in a great majority of cases, affects the lung. Its chief mode of dissemination amongst mankind appears to be by means of the dried sputum which is received immediately without the intervention of a second host. The expulsion of tubercle bacilli from the lung has been estimated by Böllinger at as many as twenty millions in twenty-four hours. Here are presented all the necessary factors to secure the continuance of the species as perfectly as though they had been expressly designed to this end.

The history of the filaria sanguinis hominis, an inhabitant of the lymphatics and blood in man, and its relation to the gnat as a carrier-host, or perhaps as an intermediate host, is full of interest. Very remarkable, too, is the adaptation of the periodic evening swarm of embryonic filariæ at the very time when the visits of the female gnat, the blood sucker, are most common. So numerous are the embryos that the smallest drop of blood is sure to contain some. The gnat, having fed, proceeds to lay her eggs in pools of water, and it must be remembered that the filaria belongs to the group of nematoda, some of which only are parasitic, while others are free water-living worms. Whether part of their life must be passed in water, in which case the gnat is merely a carrier-host, or whether they are re-introduced by the gnat in the act of biting, to which the discovery of the filaria in the proboscis of the mosquito definitely points, in either case the gnat is necessary for the continuance of the species from man to man.

Of even greater interest is the relationship of the gnat to malaria or gnat fever,[1] treated at length in the *Report of the Liverpool Malaria Expedition.* Certain kinds of gnat only, especially all species of the genus *Anopheles*, appear to be hospitable to the hæmamœbidæ, or blood parasites of this disease. In other inhospitable species ingested parasites perish within the stomach cavity, whereas in hospitable species the zygotes (the homologues of the fertilised ovum) escape from that cavity and develop in the tissues, ultimately giving rise to blasts (sexually produced progeny), which are found in the juices and salivary glands of the gnat. The fuller life-history is as follows:—

1. The youngest parasites, existing as *amœbulæ* or *myxopoda*, within the red blood-corpuscles of vertebrate host, on reaching maturity, become either—

2. *Sporocytes*, destined for asexual propagation of organisms within vertebrate host. These form *spores* (asexual progeny) by division; the spores escape into plasma, enter fresh corpuscles, become fresh *amœbulæ*, or

3. *Gametocytes*, sexual forms (male, containing many *microgametes;* female, containing one *macrogamete*). These remain unchanged in vertebrate hosts, but on entering the stomach of a suctorial insect perform sexual function, and the fertilised *macrogamete* becomes a *zygote* (the homologue of a fertilised ovum).

4. In a hospitable species the *zygote* pierces the stomach wall, affixes itself to the outer coat, and increases in size. It divides into 8—12 *meres;* each mere becomes a *blastophore*, bearing a number of filamentous *blasts* (sexual progeny), fixed to its

[1] *Report of the Malaria Expedition of the Liverpool School of Tropical Medicine and Medical Parasitology.* Memoir II. By Major R. Ross, Dr. H. E. Annett, and E. E. Austen. 1900.

surface by one extremity. The *blastophore* disappears, leaving the *zygote* packed with *blasts* (Koch's sickle-shaped bodies). The capsule ruptures, the blasts escape into the body cavity, and penetrate to the salivary glands of the gnat, whence they may pass into the blood of a new vertebrate host, in which they eventually become the amœbulæ with which the cycle commenced.

These facts, confirmed by recent experiments, thus seem to show that gnat fever is communicable from sick to healthy persons by the bite of *Anopheles*, the life-history of the parasite in its alternate generations exactly favouring this mode of propagation. In the transference of the parasites from man to man through gnats there is, the authors of the *Report* point out, an economy which would not have existed had nature sown the organisms broadcast through the soil or air on the chance of one of them occasionally infecting a human being. Probably, too, this is the only method of continuance, as it has not been shown and is difficult to imagine that the hæmamœbidæ have, in addition to their complicated life-cycle in man and gnat, any further potentialities of free life in air or in water, formerly supposed to be essential to the life-history of so-called malaria. The importance of shallow pools is, by affording suitable breeding places, to favour the continuance of the genus of gnat without which the completion of the life-cycle of the parasite would be impossible. In short, as the authors of this valuable *Report* point out, there seems to be no longer *any necessity* for believing that the parasites live at all in the air or soil of infected localities. . . . Malarial fever *is* connected with the soil, with stagnant water in rural areas, and so on, as it was supposed to be; but it is not, as was thought, the germ itself which springs from the soil, but the carrier of the germ. The fact that malarial fever is communicable from the sick to the healthy has certainly been a surprising one. It was thought that it is not communicable because it was observed that communication does not occur outside endemic areas, while within endemic areas infections were attributed to an endemic poison, which were really due to communication from the sick, *viâ* the gnat. By endemic (malarious) areas we now understand simply areas which are infected by the communicating agent (anopheles).[1]

This process of the development of the organism of gnat fever is suggestive of that undergone by the embryo of the sheep fluke.

1. The eggs, deposited near water, give rise to a ciliated free-swimming *embryo*. 2. The embryo penetrates the body of a fresh-water snail, where it becomes a *sporocyst*. 3. Within the *sporocyst* a large number of intermediate forms *rediæ* are produced; these leave the sporocyst and penetrate to the liver of the snail. 4. Within the *rediæ* small tailed *larvæ* or *cercariæ* are formed; passing out of the body of the snail they attach themselves to the stalks of water plants or stems of grass on damp ground. 5. They become encysted and are eaten by sheep, to repeat the cycle of existence.[2]

It may be urged that too great stress is at present being laid upon the *microbic origin* of the communicable diseases, tending to the obscuration of those great first desiderata—pure air, pure soil, pure water, a clean environment. But the essence of preventive medicine is to oppose the continuance of parasitic species by the production of conditions unfavourable to their existence, and so to assist the constant tendency of such species to die out, and these desiderata must ever hold the first place as prime necessaries in this endeavour. While all these great first principles of sanitation are most desirable, the correlation of special insanitary conditions to special forms of disease established by study of the life-history of the organism, must always be kept in mind; thus, excellent though the cleansing of pigstyes may be as a principle, it is but wasted energy, in the presence of a milk-carried epidemic of enteric fever, to start a crusade against pigstyes, or in the presence of spreading small-pox, to concentrate reconstructive energies upon the sewers. And the connotation of that much-abused word "insanitary" includes much beyond the mere filth conditions included in its popular conception; it includes social habits not only tending to overcrowding, but leading to free intermixing, increased by the

[1] *Op. cit.*, p. 34 *et seq.*

[2] A. P. Thomas, quoted by Payne, *Op. cit.*, p. 554.

very human impulse of curiosity or pity in times of sickness, and resulting under these influences, tinctured by a semi-religious sanction, in free visiting after death, or in the holding of disastrous wakes. Such social habits often supply the necessary facilities for widespread communication of disease, and may justly be classed as insanitary.

But the interests involved in Public Health or State Medicine in the present day are far wider than those concerned only with control of the communicable parasitic diseases. They are concerned with the conditions of workers in factories and the conditions of dwellers at home, and they take account of all influences affecting injuriously the bodily, or in the wider acceptation of State Medicine, the mental condition of the population. They are particularly concerned with the daily life and work of those classes least able to protect themselves, and most exposed, by necessities of occupation or social condition, to injurious influences. This quite modern development of Public Health has come into existence during the century preceding the present reign, and has resulted from three causes —the awakening of medical interest in questions affecting the Public Health, the development of the altruistic spirit in public policy, and the rise of and assumption of a position of numerical and political importance by the artisan classes. The revival of scientific medicine in the 17th century was followed in the 18th by the work of many pioneers who helped to establish Public Medicine upon a broad and intelligent basis. The year 1720 was signalised by the publication of Dr. Richard Mead's *Short Discourse concerning Pestilential Contagion*, which not only passed through nine editions in his lifetime, but stands even now as a remarkable essay in practical preventive medicine. In 1752 Sir John Pringle published his work on *Diseases of the Army*, which, dealing as it did with the causes and prevention of disease, as well as with the general hygienic conditions of active service, may be said to have begun hygienic reform for the army.[1] A similar service towards the navy and mercantile service was performed by Dr. James Lind, who between 1753 and 1757 published his treatise on Scurvy and his essay on the

[1] Sir John Simon, *English Sanitary Institutions*, 2nd Ed., 1897.

means of preserving the health of seamen. The awarding of the Copley Medal in 1776 to Captain Cook for successful application of these principles during a voyage round the world, and the issuing of lemon juice to the navy in 1796, during Sir Gilbert Blane's term of office on the Medical Board, form notable evidences of progress. Sir George Baker in 1767 had meanwhile demonstrated the "Cause of the Endemial Colic of Devonshire" by a remarkable and sound piece of reasoning. Finally Edward Jenner endowed the close of the century with a discovery, the far-reaching influence and value of which it is unnecessary to dilate upon and difficult to over-estimate.

Apart from medical progress, a change was taking place in the social relations of the governing and the governed classes. The care that is now exercised for the welfare of the poorer classes of citizens was, before the middle of the eighteenth century, hardly realised as possible or even necessary. The "New Humanity" of the eighteenth century was marked by two waves of enthusiasm, the altruistic political revival of the last quarter of the century having been prefaced and, no doubt, largely influenced by the remarkable religious revival of the second quarter of the century, associated with the names of Whitefield and the Wesleys.[1]

"Poverty," says Sir John Simon, "began to be considered, as perhaps never before, by the prosperous parts of society . . . The study of the various factors which are degradatory in social life were seen to be more urgent religious problems than some which had exercised schoolmen and mystics; and vagrancy and vice and crime, when the conditions of their multitudinous production had grown to be better understood, were felt to be piteous appeals to the strong of the world, brothers' blood crying from the earth." Philanthropic activity, suspended during two centuries of ecclesiastical and civil unsettlement, began to be re-devoted to the establishment of new hospitals for the sick and refuges for the destitute and afflicted, or, like the Sunday schools established by Raikes, for the elementary education of children of the poor. Foremost

[1] I am reminded in this connection of Wesley's aphorism, "Cleanliness is indeed next to Godliness," Sermon XCIII. on Dress.

amongst work bearing on the sanitary and moral relations of preventive medicine stands the memorable Enquiry of John Howard into the condition of prisons, the results of which he disclosed to the English House of Commons in 1774; and it is characteristic of Howard's singleness of purpose that, not content with generalities in humanity for mankind at large, he did his first work of sanitary reform at home, pulling down and rebuilding every one of his own cottages, and such others as he could purchase, and erecting others, so as to transform the whole village from a condition of neglect to one of cheerful well-being. The question of prison reform was taken up earnestly by other philanthropists, foremost amongst whom must be placed Mrs. Elizabeth Fry, whose influence on prison reform, especially as regards the female prisoners, has proved remarkable and enduring. Meanwhile outside events had not been without influence in modifying and directing public opinion; and the great revolutionary movements of 1776 in America and of 1789 in France profoundly influenced the line of thought, not only on abstract principles of governments, but on the reciprocal rights and duties of governors and the governed. The abolition of British slave trading in 1807, and the emancipation of Colonial slaves in 1833 supply an index of the progress and growth of the "New Humanity" in politics.

The third important factor profoundly affecting the scope and importance of Public Health measures was the change in social conditions induced by the "industrial revolution" that was a distinguishing feature of the latter half of the 18th century, the influences of which extend to the present day, and the immediate result of which was to convert Great Britain, previously, in great part, an agricultural community, into the workshop of the world. Although, early in the 18th century, the textile trades had been invaded by machinery, and improvements in process of manufacture had largely developed the iron industries, the great feature of the century was the general application of steam, by the genius of Watt, to purposes of manufacture. Dr. Russell[1] thus eloquently traces the social results of the application of steam to prime movers.

1 'Public Health and Social Problems," *San. J.*, 1898, N.S., v. 1.

"So long as the human hand is the motive power, men are not necessarily drawn together in community of labour. With the use of wind and water comes the factory system, but the factories must go where the wind and water are to be had. The hand-loom weaver lives in villages; the power-loom weaver, so long as the power is wind and water, congregates on breezy heights and beside isolated rivers, in groups which have their natural limitations. The spinster spins at home. Steam changes all that. The power is brought to the factories in the shape of coal. Manufacturing towns spring up on sites which attract, by facility of production and distribution, and grow without natural check or limit. And so with other industrial occupations. If a coalfield is convenient, they cluster round it; or a population grows up, maintained by getting and shipping or training the coal to seats of industry elsewhere. If ironstone is found in proximity to coal, they, in combination, attract the most portentous aggregations of human beings. Thus whole counties become practically urban in character. The country becomes a vast workshop. Ports are required, and spring up, from which to send out the products, where to receive and whence to distribute the merchandise that comes in exchange."

In this way was created the artisan class, which, though at first very hardly treated by the new conditions of labour, has now, under State protection, become the most numerous and energetic portion of the community. The growth of manufacturing towns takes place largely by the influx of adults from country districts, census after census shows a greater and yet greater aggregation in towns and a corresponding depletion of rural districts: and this aggregation undoubtedly presents added difficulties, and is a further distinct source of danger to public health, in no way lessened by increased facilities in travelling, and constant migration from centre to centre with the varying fortunes of local industries.

Such were the partly favourable, partly unfavourable, and wholly new conditions of social life, upon which a new epidemic invasion was, towards the close of the first third of the century, to exert a salutary stimulus. The tendencies making for improvement, forming through the previous century, were as yet barren of result in Public Health progress. Though no vast epidemic had terrorised the population, yet the home fevers and small-pox were constantly claiming an undue proportion of victims. The congestion induced by industrial aggregation in large towns furnished fresh facilities for the spread of disease, which the condition of these towns as to housing and municipal control served to aggravate. That one-tenth of the population of Manchester was at this period living in cellars, gives some idea of the urgent need for reform. Indications, such as the progress of factory reform through the first half of the century, marked chiefly by the Acts of 1833 and 1847, and indissolubly associated with the name of Lord Shaftesbury, the Reform Bill of 1832, and the Amendment of the Poor Law in 1834, show that the humanitarian and social influences were still at work; but some stimulus was needed to initiate and direct the beginnings of sanitary effort. The cholera of 1831 supplied this stimulus, and, disastrous as were its immediate effects, there is little doubt that it originated those movements which formed the beginnings of modern preventive medicine. The lessons of 1831 might have been forgotten, but the establishment of civil registration in 1837 made possible, for the first time, an accurate estimate of the loss caused by disease and death, emphasised and elucidated by the writings of Dr. Farr. Public opinion, as yet only fitfully interested towards sanitary improvement, became thoroughly aroused. In 1844 the Health of Towns Commission published their classical Report, followed by the first Public Health Act of 1848. In 1849 and 1854, cholera repeated its visits and re-emphasised its warnings, and at length an era of sanitary reform, continuous and progressive, was established. Great as were the advances made, and marvellous as were the inductions of such men as Henle, Snow, and William Budd, made from the observed facts of disease occurrence and transmission, as to the actual nature of contagion, yet it needed the actual demonstration of the "contagium," and of its causal relationship to its associated disease, to place preventive medicine on an assured basis.

The modern science of bacteriology, a product especially of the last quarter of the century, has elucidated much that was obscure in medicine, and furnished a new standpoint for accurate study of pathology, as well as of the propagation of disease. The science is yet young, but in a vigorous infancy; already it has marvellously advanced surgery, and systematised much of medicine; more than all, it has made it evident that public medicine is no alien, but a fellow-worker with the sister crafts, and that the interests of one are the interests of all.

---

### A PLEA FOR SANATORIA FOR THE POORER CONSUMPTIVES.

